# Poster Session II - A277 HIGHER BMI DOES NOT IMPAIR VISUALIZATION OF BOWEL SEGMENTS OR ANASTOMOSES IN INTESTINAL ULTRASOUND

**DOI:** 10.1093/jcag/gwaf042.277

**Published:** 2026-02-13

**Authors:** D Hazra, B Maracle, K Novak, C Lu, J Besney, R Reji, A AlDarwish, G G Kaplan, C Seow, C Ma, R Ingram, R Panaccione, J St-Pierre

**Affiliations:** University of Calgary, Calgary, AB, Canada; Medicine, University of Calgary, Calgary, AB, Canada; Medicine, University of Calgary, Calgary, AB, Canada; Medicine, University of Calgary, Calgary, AB, Canada; Medicine, University of Calgary, Calgary, AB, Canada; Division of Gastroenterology and Hepatology, University of Calgary, Calgary, AB, Canada; Medicine, University of Calgary, Calgary, AB, Canada; Medicine, University of Calgary, Calgary, AB, Canada; Medicine, University of Calgary, Calgary, AB, Canada; Medicine, University of Calgary, Calgary, AB, Canada; Medicine, University of Calgary, Calgary, AB, Canada; Medicine, University of Calgary, Calgary, AB, Canada; Medicine, University of Calgary, Calgary, AB, Canada

## Abstract

**Background:**

Intestinal ultrasound (IUS) is a non-invasive, point-of-care tool to assess disease activity in inflammatory bowel disease (IBD). Bowel wall thickness (BWT) is among the most reliable markers of active inflammation. A longstanding concern is that higher body mass index (BMI) may impair visualization due to increased abdominal wall thickness and beam attenuation, however, data quantifying the effect of BMI on IUS performance in IBD are limited.

**Aims:**

We aimed to assess whether BMI affects bowel visualization in clinical IUS practice.

**Methods:**

We retrospectively analyzed IUS scans at a tertiary IBD center (August 2021-August 2025). Successful visualization was defined by the ability to obtain a BWT measurement. Scans were acquired by three experienced bowel sonographers using standardized protocols. Rectal visualization was assessed in a predefined subgroup (n = 135). Visualization rates of bowel segment and BMI category (<18, 18–25, 25–30, ≥30 kg/m2) were compared using the Freeman-Halton test.

**Results:**

748 scans from 516 patients (median age 43 years [IQR 32-59], 50% female, median BMI 24.9 [22.1–28.6]) were included. Among those with native anatomy (n = 589), the terminal ileum (TI), colonic segments, and rectum were visualized consistently across BMI groups with no significant difference. Among patients with prior ileocecal resection (n = 159), there was no significant difference in anastomosis visualization across BMI groups (p = 0.051). In patients with ileal Crohn’s disease (n = 269), TI visualization was >93.6% across all BMI groups (p = 0.63). In IBD patients without ileal disease (n = 318), visualization did not differ significantly across BMI groups (p = 0.15). However, a significant difference was observed in the overweight group between IBD cases involving the TI and those sparing it (97.7% vs 85.5%, p = 0.005). Analysis using only the last scan per patient (n = 516) confirmed these findings.

**Conclusions:**

IUS visualization of bowel segments and anastomoses was consistent across BMI categories. Visualization was more influenced by disease location than BMI, underscoring the robustness of IUS across body habitus.

A277 Table 1: IUS Visualization by BMI Category

**Funding Agencies:**

None, NRC

**Table:** 

BMI Category	Ileal Crohn’s (n = 269) n (%)	Non-TI disease (n = 318) n (%)	p-value	Anastomosis Visualization (n = 159) n (%)
<18	9/9 (100)	5/5 (100)	n/a	8/8 (100)
18-24.9	121/127 (95.3)	144/153 (94.1)	0.79	59/73 (80.8)
25-29.9	84/86 (97.7)	77/90 (85.5)	0.005	51/56 (91.1)
≥30	44/47 (93.6)	63/70 (90.0)	0.74	15/22 (68.2)
p-value	0.63	0.15		0.051

